# T121. RATES AND PREDICTORS OF RELAPSE FOLLOWING DISCONTINUATION OF ANTIPSYCHOTIC MEDICATION AFTER A FIRST EPISODE OF PSYCHOSIS

**DOI:** 10.1093/schbul/sby016.397

**Published:** 2018-04-01

**Authors:** Meghan Bowtell, Scott Eaton, Kristen Thien, Melissa Bardell-Williams, Linglee Downey, Aswin Ratheesh, Eoin Killackey, Patrick D McGorry, Brian O’Donoghue

**Affiliations:** Orygen, the National Centre of Excellence in Youth Mental Health

## Abstract

**Background:**

There is uncertainty about the required duration of long-term antipsychotic maintenance medication after a first episode of psychosis. Robust predictors of relapse after discontinuation are yet to be identified. The present study aimed to determine the proportion of young people who discontinue their antipsychotic medication after a first episode of psychosis, the proportion who experience relapse, and predictors of relapse.

**Methods:**

A retrospective study of all individuals presenting to the Early Psychosis Prevention and Intervention Centre between 01/01/11 and 31/12/13 was conducted. A Cox regression analysis was conducted to identify predictors of relapse.

**Results:**

A total of 544 young people with a FEP were included. A trial of discontinuation was undertaken by 61% of the cohort. Median duration of antipsychotic medication prior to first trial of discontinuation was 174.50 days. Amongst those trialing discontinuation, 149 (45.8%) experienced relapse in a median follow-up time post discontinuation of 372 days. On multivariate analysis, predictors of relapse were a diagnosis of cannabis abuse disorder (HR: 1.40), and longer duration of antipsychotic medication (HR: 1.05).

**Discussion:**

Antipsychotic discontinuation frequently occurs earlier than guidelines recommend. Individuals with a diagnosis of cannabis abuse are more likely to experience relapse and addressing this substance abuse prior to discontinuation could possibly reduce relapse rates.

